# A Comparison of the Plyometric Performance of Upper Limbs between Experienced and Non-Experienced Athletes

**DOI:** 10.3390/sports12080217

**Published:** 2024-08-12

**Authors:** Sylvain Dhote, Pauline Eon, Sidney Grosprêtre

**Affiliations:** 1 Laboratory of C3S (Culture, Sport, Santé, Société) (EA4660), 25000 Besançon, Francesidney.grospretre@univ-fcomte.fr (S.G.); 2 Institut Universitaire de France (IUF), 92073 Paris, France

**Keywords:** street workout, push-ups, plyometrics, concentric, electromyography, echography

## Abstract

Although explosive upper-limb movements are far less studied than the equivalent lower-limb movements, they are important in many sports activities. The goal of this study was to explore, for the first time, the performance of street workout (SW) athletes who primarily focus on explosive and isometric strength in the upper limbs and to examine the effect of the contraction type on performance during a classical upper-body movement. Eighteen men took part in this study (age: 22.6 ± 2.1 years, height: 179.6 ± 7.1 cm, bodyweight: 71.9 ± 6.6 kg). Of these, nine practiced SW and nine practiced various team and individual sports—the latter serving as the control group. The athletes performed three different types of push-ups—one in a concentric way and two others in a plyometric way—and a fatigue-inducing push-up set. Jump heights, myoelectrical activities (through electromyography), muscle architecture, and hypertrophy (through ultrasonography) were measured. The results show no significant differences in jump height between the push-up types. Both groups confounded, but the SW athletes jumped on average 66 ± 21% higher than the control athletes (*p* < 0.05). There was no major difference in EMG between groups, regardless of the push-up type, but the SW athletes had a greater pectoralis major and anterior deltoid thickness as well as a greater pennation angle of the pectoralis major. The results suggest that the plyometric performance of the upper limbs does not follow the same pattern as that of the lower limbs. The SW group’s greater capacity in performing explosive push-ups could be attributed to greater upper-body muscle hypertrophy and more efficient execution of the movement.

## 1. Introduction

Classically, dynamic exercises are exercises that imply changes in muscle length. Concentric change refers to a shortening of muscle, and eccentric change refers to a lengthening, and when using a stretch–shortening cycle, the quick succession of both of these changes results in what we call a plyometric contraction [1]. For several decades, scientific research has extensively studied explosivity and plyometrics in the lower limbs. Haff and colleagues defined explosive exercises as “produc[ing] a maximal or near maximal initial rate of force development that is maintained throughout a specified range of motion” [2].

As mentioned above, studies on plyometrics have mostly investigated the lower limbs. These studies include classical squat jumps [3,4], which are achieved by performing the descent phase of a squat, pausing the movement for 2 s when the legs are parallel to the ground, and then performing the ascent phase as fast as possible in order to jump as high as possible. There are also countermovement jumps, which are achieved by performing a squat jump without stopping the movement [5]; ankle hops, where only the ankle produces the jump [6]; single leg hops [3,7]; and some others [8,9]. On the other hand, there have been very few studies of upper-limb explosive movements [1] performed using only one’s bodyweight. According to Koch, Riemann, and Davies [10], the majority of studies on upper-limb explosive movements have dealt with power development using open kinetic chain exercises, such as medicine ball throws [6,11], as well as movements involving throws above the head [12,13,14]. There seems to be a lack of scientific research on plyometrics in closed kinetic chain upper-body exercises. Upper-limb explosive movements are particularly common in some sports, including team sports, such as handball [15] and basketball, and individual sports, such as weight throwing and artistic gymnastics. Therefore, it is necessary to investigate how plyometric exercises using the arms can help performance in these sports and, on the contrary, how athletes in these sports perform plyometric tests using the arms. 

Several studies have examined the test–retest reliability of the parameters of force on plyometric push-ups on force plate exercises in order to assess upper-body power [16,17,18,19]. The results of the most recent study [17] suggest that the parameters obtained by force plates when performing plyometric push-up exercises represent a valid and reliable tool for assessing upper-body power. Aside from studies on the reliability of push-up tests, few studies have examined how variations in push-ups affect force parameters. For example, ref. [10] showed that during different plyometric push-ups, the loading and propulsion rates were higher for “clap push-ups” than for drop push-ups from different heights. A recent systematic review [20] examined all studies presenting kinetic data on 46 push-up variations. Out of 26 studies, only 3 presented the flight time in push-ups [16,21,22], and only 1 investigated the effect of the push-up modality on jump height [21]. 

To our knowledge, very few studies have investigated whether an increase in jump height between concentric and plyometric push-ups is similar to the increase observed between squat jumps and countermovement jumps, particularly in athletes who are already trained in these jumps. One study [23] showed that the difference between squat jump and countermovement jump heights depended on the sport practiced. For example, short-distance runners had the highest jump values, and long-distance runners had the lowest. In this model, we aimed to verify whether the maximal height reached by performing explosive push-ups varies between athletes from different sports. Additionally, several studies have shown that explosive push-up variations are useful and valid exercises for assessing upper-body power [16,20]. In a recent study [19] assessing the validity of five push-up variations to evaluate upper-body power, the exercises chosen were derived from explosive exercises (e.g., squat jumps, countermovement jumps, and drop jumps) used to develop and assess lower-limb explosiveness. In the present study, very similar variations of these push-ups were used—namely, standard countermovement push-ups (referred to as “plyometric push-ups” in the present study), standard squat push-ups (“concentric push-ups”), and drop-fall push-ups (“drop push-ups”). There were small differences in the execution of these modalities. The instructions for the execution of these three modalities are described in the methods section.

The upper-limb training model chosen for this study was street workout (SW). SW is an emerging and growing sport in which athletes perform strength movements on gymnastics apparatus and use their upper limbs almost exclusively to support their whole body. Among the most important physical qualities to possess for performing SW are the maximal strength of the upper body and, more precisely, the strength-to-weight ratio—that is, the maximal strength in a general weightlifting exercise (in kg) or a specific SW exercise divided by the mass of the body (in kg). This ratio, despite not being widespread in the SW community, is a critical aspect of this sport—as it is for gymnastics and climbing—as athletes have to easily overcome their bodyweight in their respective environments. During the development phase in SW, it is common to practice arm plyometric training. For example, athletes practice plyometric push-ups as well as plyometric dips and handstand push-ups. However, classical SW upper-body training is also markedly isometric. Indeed, one-third of performances in competitions are called “statics” and consist of isometric holds of a particular body position on an apparatus over 2–5 s or sometimes more. It is therefore of interest to verify whether this particular means of training—consisting of balanced isometric and dynamic contractions—surpasses that of athletes from other sports, as some coaches and athletes still doubt the efficiency of SW training for building muscle, strength, and power. If this approach is efficient in these aspects, to what exact extent is it so, given that there are little to no data on the actual performance of SW practitioners? Indeed, to date, only studies conducted on SW athletes have focused mainly on anthropometric data and specific performances (handgrips) [24,25]. The aim of the present study is twofold. The first aim is to compare the plyometric performance in different types of push-ups executed on the same model as squat jumps, countermovement jumps, and drop jumps on the legs. The second aim is to investigate possible differences between experienced and unexperienced athletes. We formed the following hypotheses: (1) Plyometric performance on the upper limbs follows the same behavior as on the lower limbs and (2) SW athletes perform better in plyometric push-ups than other athletes with similar training volumes.

## 2. Materials and Methods

### 2.1. Study Design

This study took a cross-sectional approach, presenting a comparative analysis of the performance of SW athletes and athletes of other sports in several types of push-ups. This was a quasi-experimental study as some external variables related to the training characteristics of the participants were not controlled. The 18 participants in this study all performed three different kinds of push-ups, which were chosen to match the contraction modalities of the classical jumping exercises: concentric push-ups (CONC) for squat jumps (SJ), plyometric push-ups (PLYO) for countermovement jumps, and drop push-ups (DROP) for drop jumps. Jump height was measured as an indicator of upper-body muscle power and was compared between the push-up modalities. Electromyographic activity was measured as an indicator of neuromuscular activity, and muscular ultrasonography was performed to assess muscle characteristics as an indicator of peripheral development. 

### 2.2. Participants

Eighteen young men participated in this study (age [mean ± SD]: 22.6 ± 2.1 years, height: 179.6 ± 7.1 cm; bodyweight: 71.9 ± 6.6 kg), with nine participants in the CO group and nine in the SW group. For the SW group, age was 22.7 ± 2.7 years, height was 178.6 ± 6.5 cm, and body weight was 72.7 ± 5.2 kg. For the control group (CO), age was 22.4 ± 1.5 years, height was 180.6 ± 7.9 cm, and body weight was 71.1 ± 8.0 kg. Independent sample *t*-tests conducted on the age, height, and weight of both groups showed no significant differences. The CO participants were athletes from various team and individual sports (two from football, two from weightlifting, one from triathlon, one from CrossFit, one from parkour, one from basketball, and one from rugby). The SW participants had all practiced SW and/or calisthenics for at least 1 year, although only 3 regularly took part in competitions. In terms of explosive push-ups, the SW group constituted the experienced athletes, while the CO group constituted non-experienced athletes. 

The inclusion criteria were as follows. The participants had to be male and at least 18 years old. Each athlete had to have spent at least 1000 h practicing their own sport. Members of the SW group had to have practiced regularly over the previous 2 years and followed a traditional SW training plan, including all of the specific pushing exercises. Members of the CO group had to have practiced regularly over the previous 2 years. They had to be accustomed to general strength and conditioning for their sport, but they were not specifically trained in PU exercises. The exclusion criteria were as follows. Participants who were injured or who could not safely perform the push-up tests were not included. For the CO group, they could not be experienced with explosive push-ups. In addition, they should not have taken part in any intense training 48 h prior to the tests. They should have had at least 6 h of sleep during the night prior to the tests and should not have taken any stimulating substances, such as caffeine, 3 h prior to the tests.

The study protocol was conducted in accordance with the ethical principles of the Declaration of Helsinki of 1983 and approved by the regional ethics committee (Comité d’Ethique pour la Recherche de l’Université Bourgogne Franche-Comté, no. CERUBFC-2022-04-25-013). All participants provided written informed consent to participate in the study.

### 2.3. Procedures

The participants visited the laboratory once for an experimental session of about 1 h.

The experimental protocol was as follows. While each participant was sitting, the experimenter performed muscular echography on four different sites: the pectoralis major (PEC), the anterior deltoid (DELT), the long head of the triceps brachii (TRI LONG), and the lateral head of the triceps brachii (TRI LAT). From this, lean muscle thickness and pennation angle measurements were recorded (the distance between the bone and adipose tissue and the average angle between the muscle fascicle and the mid-muscle aponeurosis). 

The participants then performed a standardized warm-up. This lasted 10 min, including 3 min of jogging and 2 min of articular warm-up, followed by 2 sets of 5 repetitions of slow push-ups and 2 sets of 5 repetitions of pressure and stretching of the wrist against each other. The participants then had a 2-min rest while the 4 electromyographic (EMG) sensors were placed on the pectoralis major, anterior deltoid, and lateral and long head of the triceps brachii muscle after their skin had been dry-shaved and cleaned with alcohol. An accelerometer was placed behind their necks, and accelerations were recorded at 2000 Hz across three dimensions.

For the subsequent tests, the arm jump height and EMG were recorded for each repetition. Each participant had to perform 2 repetitions of explosive push-ups in the CONC modality; 2 repetitions in the PLYO modality; 2 repetitions in the DROP modality; and 20 repetitions in the PLYO modality, with no interruptions between the repetitions. The order in which the participants performed the first three modalities of push-ups (CONC, PLYO, and DROP) was randomized. The fourth modality was always a set of 20 repetitions. There was 2 min rest between each modality and 1 min rest between each repetition (except for the fourth modality).

#### Upper-Limb Performance

**Figure 1:**
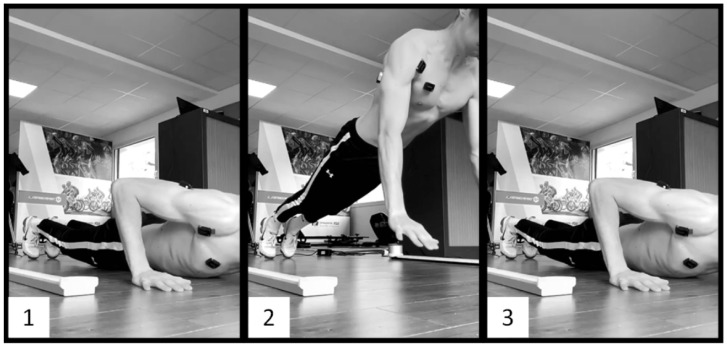
Concentric push-up. 1: Starting position: chest touching the ground. 2: Flight phase: arms extended. 3: Ending position: landing on the palms.

**Figure 2:**
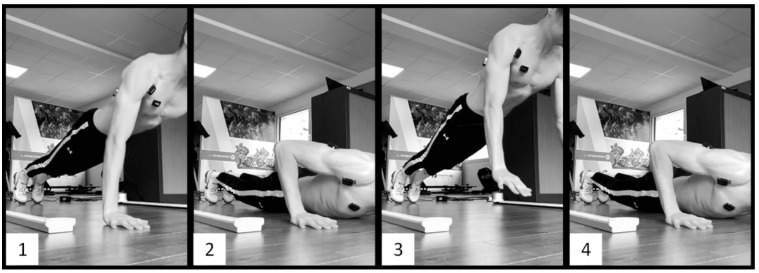
Plyometric push-up. 1: Starting position: arms extended. 2: Descent phase: arms bending rapidly. 3: Flight phase: occurring after a quick eccentric-to-concentric muscle contraction. 4: Ending position: landing on the palms.

**Figure 3:**
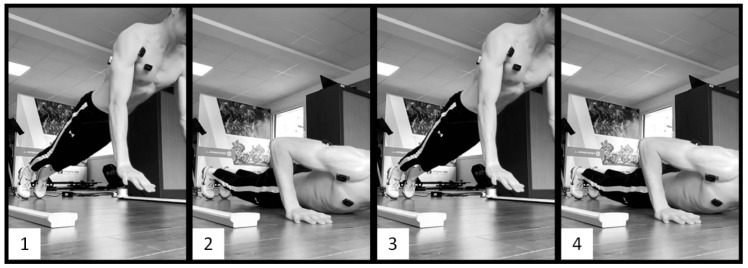
Drop push-up. 1: Starting position: landing from an elevated support. 2: Descent phase: arms bending rapidly. 3: Flight phase: occurring after a quick eccentric-to-concentric muscle contraction. 4: Ending position: landing on the palms.

For the three above-mentioned types of push-ups, the goal was for the participants to jump as high as possible while extending their arms as quickly as possible. The CONC push-ups consisted of performing only the ascent phase of the push-ups, starting from the bottom position with the chest on the floor and utilizing only concentric muscle contractions (Figure 1). The PLYO push-ups consisted of performing regular push-ups with a descent and an ascent phase without interruption, utilizing both eccentric and concentric—and thus plyometric—contractions (Figure 2). The DROP push-ups consisted of performing PLYO push-ups with a higher starting position. The participants had to place their hands on two 12 cm-high wooden blocks and then push slightly on the blocks to let themselves fall and land with their hands on the floor, directly under their shoulders. Without interruption, they finished with the ascent phase of the push-up (Figure 3). 

The experimenter visually examined the validity of each push-up. The verbal instruction was as follows: “Perform a push-up in order to jump as high as possible.” If the participants made a mistake, they were immediately told to stop and had to restart after verbal instructions to detail the corrections were given. The correct execution of the PLYO push-ups was as follows: The head, trunk, and legs were fixed together, and the shoulders, pelvis, and ankles were always aligned. The participants were asked to place their hands just under their shoulders, with their arms vertical when straight. This instruction implied that the width between both hands varied for each participant as it depended on each participant’s shoulder width. This allowed each participant to be in a comfortable position when performing the push-ups. Finally, when viewed from the top, the arms had to form an angle of 45° or less with the trunk. 

### 2.4. Ultrasound Analysis

The participants lay in a relaxed sitting position for 10 min before any measurements were taken. A 5.5 cm (7.5 MHz) linear array probe (Mindray, Shenzhen, China) was positioned perpendicular to the dermal surface and oriented along the longitudinal axis of the muscle–tendon unit. The pectoralis major, anterior deltoid, and long head and lateral head of the triceps brachii were measured on the right side of each participant. For the pectoralis major, the probe was placed at one-third of the distance between the insertion of the pectoralis on the humeral bone and the insertion on the sternum. For the anterior deltoid, the probe was placed 4 cm distal and anterior to the acromion. For the lateral head of the triceps brachii, the probe was placed 2 cm laterally at 50% of the distance of the line from the olecranon to the acromion. This was the same for the long head, except that the probe was placed 2 cm medially.

Two images of each muscle were recorded using B-mode Zonare ultrasound video imaging on a portable echograph (Mindray, Shenzhen, China). Muscle thickness (MT) and pennation angles (PA) were extracted. The criteria for storing the images were parallel superficial and deep aponeurosis and the presence of at least three discernible fascicles with their junction upon deep aponeurosis. On each image, MT was measured from the average distance between the deep and superficial aponeurosis, measured directly by ultrasound software. PA was measured from the average angle between the muscle fascicle and the mid-muscle aponeurosis.

### 2.5. Electromyography

EMG activity was recorded from three upper limb muscles on the right arm (PEC, DELT, and TRI LAT). The skin was first dry-shaved and cleaned with alcohol to keep low impedance (<5 kΩ). EMG signals were recorded with Trigno sensors (Delsys, Natick, MA, USA) firmly strapped to the arm with a skin rubber. Sensors were placed according to SENIAM recommendations [26]. EMG signals were amplified with a bandwidth frequency ranging from 0.3 Hz to 2 kHz (gain: 1000) and digitized online (sampling frequency: 2 kHz) using Labchart software (LabChart 8, ADInstruments, Sydney, Australia).

A fourth sensor identical to the first was placed behind the participants’ necks to record the linear acceleration on the axes of the three dimensions. 

### 2.6. Data Analysis 

The percentages of the relative jump heights during the 20 repetition sets were calculated as follows: (jump height reached at each repetition)/(height reached at the first repetition) × 100. The raw EMG data were filtered by root mean squared (RMS) using Labchart software (LabChart 8, ADInstruments, Sydney, Australia). The normalized signal of the RMS of EMG, as a percentage, during each of the three push-up modalities was calculated as follows: (amplitude of RMS of EMG on the modality)/(maximal amplitude of RMS recorded) × 100. The normalized signal of the RMS of EMG during the 20 repetitions set was calculated as follows: (amplitude of RMS of EMG on the repetition)/(maximal amplitude of RMS recorded in the set) × 100.

### 2.7. Statistical Analysis 

Tests were conducted to examine the potential differences in performance between the two groups of athletes (SW and CO) and to examine the differences between the various types of push-ups. All data are presented as mean ± standard deviation. The normality of the data and homogeneity of the variances were tested using the Shapiro–Wilk test and Levene’s test, respectively. Separate analyses were performed for the data on the jump heights and the RMS of the EMG. The data were analyzed by means of an analysis of variance (ANOVA), with the two factors being the group factors (SW and CO) and the modality factors (CONC, PLYO, and DROP). The main effects and interactions were followed up using Tukey’s post hoc HSD test. For the MT measurements, independent sample *t*-tests were conducted to compare both groups. Independent sample Mann–Whitney tests were conducted to compare groups on the relative jump height evolution. Independent Welch tests were conducted to compare the mean EMG ratios during the long push-up sets. Tests of correlation using the Pearson test were used to examine the relationship between the indicator of upper-body power and the MT of the participants. Statistical analysis was performed using JASP Version 0.16.3 software (JASP Team, 2022). Statistical significance was set at a level of 0.05. 

## 3. Results

### 3.1. Concentric, Plyometric, and Drop Push-Up Performances

**Figure 4:**
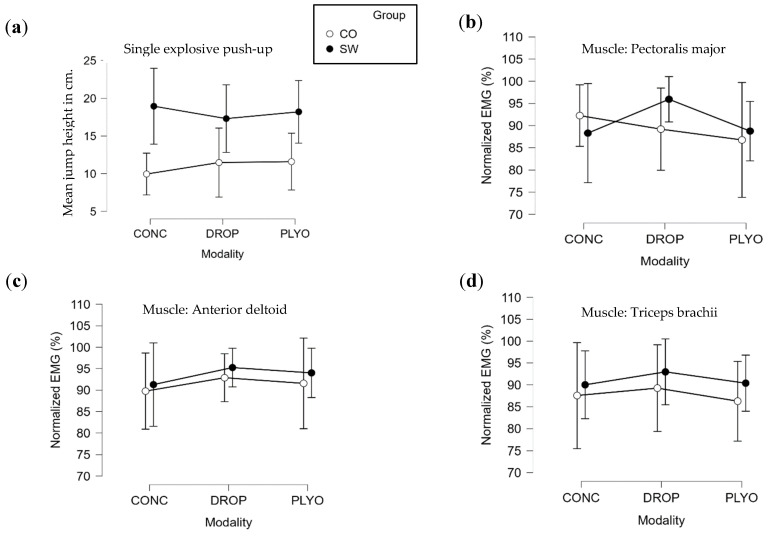
Results of the three modalities of push-ups. (**a**) Performance of the three modalities of push-ups. (**b**) Pectoralis major EMG during push-ups. (**c**) Anterior deltoid EMG during push-ups. (**d**) Triceps brachii EMG during push-ups. CONC: concentric, PLYO: plyometric, DROP: drop, ●: SW group, ○: Control group. Error bars represent 95% confidence intervals and are displayed on each graph.

The group factor had a significant effect on jump height during push-ups [F (1, 17) = 23.171, *p* < 0.001, η^2^ = 0.321]. The modality factor had no significant effect on jump height [F (2, 34) = 0.047, *p* = 0.954, η^2^ = 0.001], and there was no interaction effect between group and modality on jump height [F (2, 34) = 0.413 *p* = 0.664, η^2^ = 0.011] (Figure 4a).

The post-hoc tests conducted on the group factor showed that the mean SW jump height was significantly greater than the mean CO jump height (18.1 ± 5.7 and 11.0 ± 4.8 cm, respectively; *p* < 0.001).

The mean jump height for the CONC push-ups was 9.9 ± 3.6 cm for the CO group and 18.9 ± 6.5 cm for the SW group. For the PLYO push-ups, it was 11.4 ± 6.0 cm for CO and 17.3 ± 5.8 cm for SW. For the DROP push-ups, it was 11.6 ± 4.9 cm for CO and 18.2 ± 5.4 cm for SW (Figure 4a).

### 3.2. Linear Acceleration 

There was no significant effect of the modality factor [F (2, 13) = 2.227 *p* = 0.128, η^2^ = 0.045] and no interaction effect of group × modality [F (5, 13) = 0.076, *p* = 0.927, η^2^ = 0.002] (for CONC: Acc = 2.94 ± 0.27 G; for PLYO: Acc = 2.99 ± 0.29 G; for DROP: Acc = 3.06 ± 0.26 G).

### 3.3. Myoelectrical Activity

There was no significant effect of group [F (1, 17) = 0.250, *p* = 0.619, η^2^ = 0.005], modality [F (2, 34) = 0.775, *p* = 0.467, η^2^ = 0.031], or group × modality [F (2, 34) = 0.932, *p* = 0.401, η^2^ = 0.037]. On the DELT muscle, there was no significant effect of group [F (1, 17) = 0.593, *p* = 0.447, η^2^ = 0.012], modality [F (2, 34) = 0.564, *p* = 0.573, η^2^ = 0.023], or group × modality [F (2, 34) = 0.011, *p* = 0.989, η^2^ < 0.001]. On the TRI muscle, there was no significant effect of group [F (1, 17) = 1.137, *p* = 0.292, η^2^ < 0.024], modality [F (2, 34) = 0.286, *p* = 0.752, η^2^ < 0.012], or group × modality [F (2, 34) = 0.024, *p* = 0.976, η^2^ = 0.001]. The results are shown in Figure 4b–d for PEC, DELT, and TRI, respectively. 

### 3.4. Long Push-Up Sets

On average, the SW athletes jumped significantly higher than the CO athletes during the long set of 20 push-ups (*p* < 0.001). The mean values for the 20 repetitions were 11.0 ± 5.8 cm (SW) and 5.4 ± 3.5 cm (CO).

On average, the SW athletes had significantly higher jump heights than the CO athletes when compared with the first jump height (*p* < 0.001). The mean ratio of jump height reached was 92.2 ± 22.8% for SW athletes and 74.9 ± 29.9% for CO athletes.

**Figure 5:**
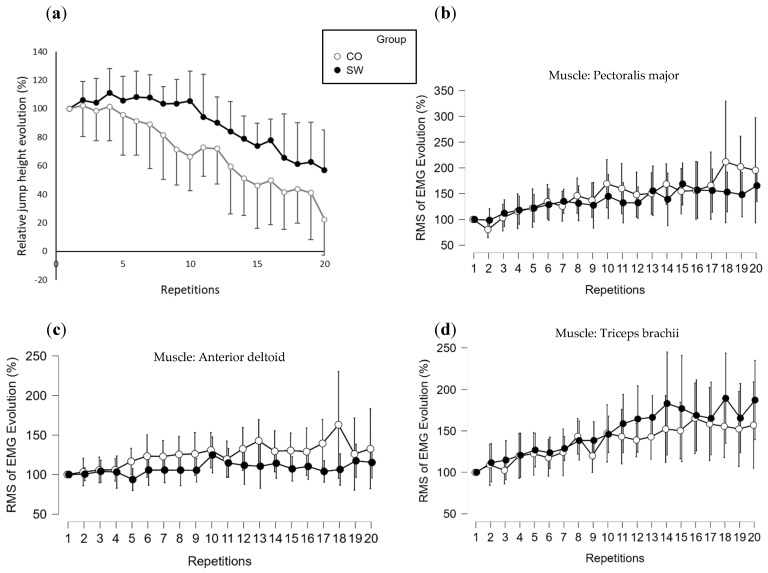
Results for the set of 20 push-up repetitions. (**a**) Performance for the set of 20 push-up repetitions. (**b**) Pectoralis major EMG during the set of 20 push-up repetitions. (**c**) Anterior deltoid EMG during the set of 20 push-up repetitions. (**d**) Triceps brachii EMG during the set of 20 push-up repetitions. RMS of EMG: maximal amplitude of the signal of root mean squared of electromyography. ●: SW group, ○: Control group. Error bars represent 95% confidence intervals and are displayed for each graph.

Figure 5a shows the results obtained for the SETMAX relative jump height evolution, with the percentage of height reached for reach repetition on the vertical axis relative to the height reached for the first repetition. 

The following were the mean ratios of EMG (i.e., the mean of the maximum RMS of EMG of each repetition divided by the RMS of EMG of the first repetition): PEC–CO: 146 ± 67%; PEC–SW: 136 ± 49% (no significant difference); DELT–CO: 125 ± 38%; DELT–SW: 108 ± 23% (DELT–CO > DELT–SW, *p* < 0.001); TRI–CO: 136 ± 42%; and TRI–SW: 149 ± 52% (TRI–CO < TRI–SW, *p* = 0.011) (see Figure 5b–d for PEC, DELT, and TRI, respectively).

### 3.5. Muscle Architecture

**Figure 6:**
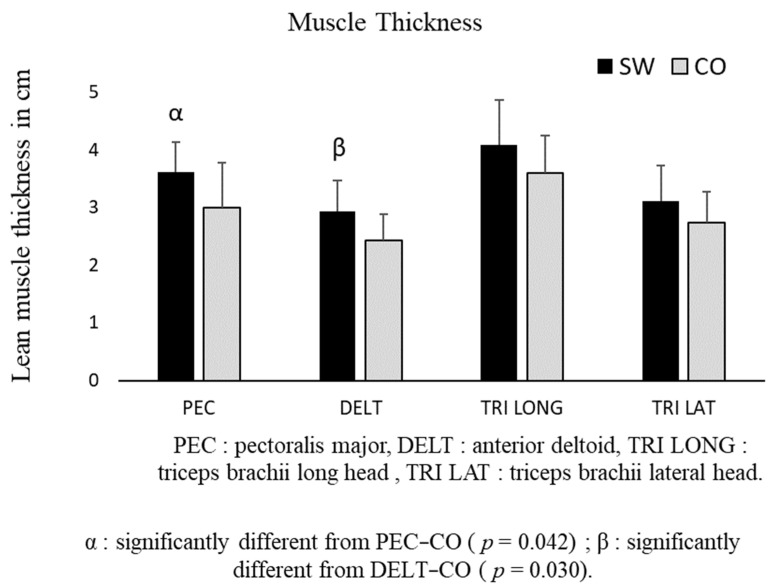
Comparison of thickness of four muscles groups involved in push-ups for the SW and CO groups. 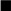 SW group, 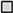 CO group, α and β: degree of significant difference. Error bars represent standard deviations and are displayed on the graph.

The independent *t*-tests conducted on the means of lean MT revealed that there were differences between the SW and CO groups for the PEC and DELT muscles (*p* = 0.042 and *p* = 0.030, respectively) (Figure 6). The independent *t*-tests conducted on the means of the PAs revealed that there were no differences between the SW and CO groups overall. However, there was a significant difference for PEC (CO: 3.7 ± 2.3°, SW: 6.9 ± 2.9°; *p* < 0.05). No significant difference was observed between the CO and SW groups for DELT (CO: 9.3 ± 3.4°, SW: 9.4 ± 4.3°), TRI LONG (CO: 14.8 ± 3.6°, SW: 19.3 ± 9.0°), or TRI LAT (CO: 14.6 ± 7.5°, SW: 17.4 ± 7.4°).

### 3.6. Relationships between Variables

**Figure 7:**
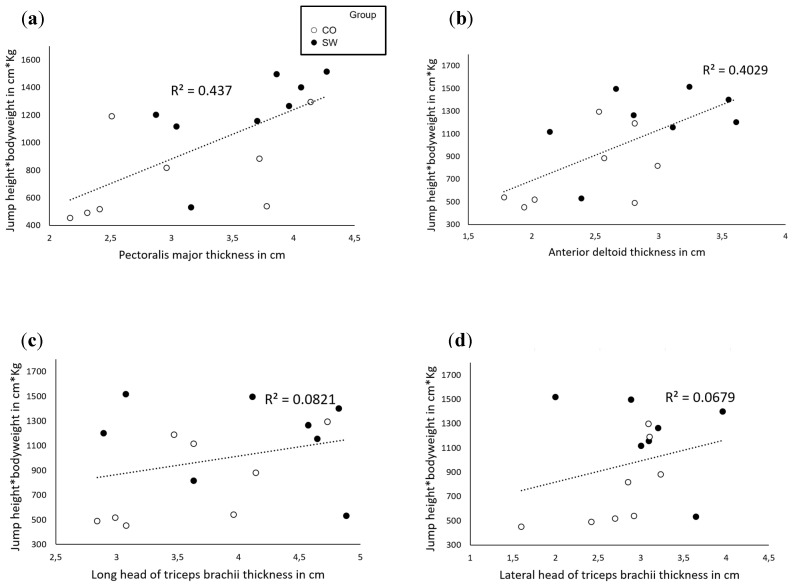
Graphs showing correlations between muscle thickness and an indicator of upper-body power. (**a**) Correlation between upper-body power and pectoralis major thickness. (**b**) Correlation between upper-body power and anterior deltoid thickness. (**c**) Correlation between upper-body power and long head of triceps brachii thickness. (**d**) Correlation between upper-body power and lateral head of triceps brachii thickness. ●: SW group, ○: Control group. Dotted lines represent the linear relationship between the indicator of upper-body power and muscle thickness. Coefficients of correlation (r^2^) are displayed in each graph.

Correlation tests were conducted to see if there was any correlation between muscle size and the ability to produce power (Figure 7). An indirect indicator of power was used—namely, the jump height in cm multiplied by the bodyweight of the athlete in kg. Pearson correlation tests revealed that there was a positive significant moderate correlation between the pectoralis major thickness and height × bodyweight (r = 0.661, *p* = 0.003) as well as between the anterior deltoid and height × bodyweight (r = 0.635, *p* = 0.004). There was no significant correlation between the long and lateral head of the triceps brachii and height × bodyweight (r = 0.287, *p* = 0.141 and r = 0.261, *p* = 0.174, respectively). 

## 4. Discussion

The aim of this study was to compare (1) the performance across three different modalities of push-up exercises as well as a long, fatigue-inducing set of push-ups and (2) the performance of SW athletes compared with other athletes with similar training volumes. The chosen modalities resembled the modalities of three lower-limb explosive exercises: squat jumps, countermovement jumps, and drop jumps. The results show that, overall, SW athletes had greater jump heights than CO athletes. However, there were no differences between the modalities of push-ups regarding jump height and EMG measurements. 

SW athletes spend a much larger percentage of time training their arms than athletes in other disciplines because of the nature of the sport and the necessity of building upper-limb strength to perform well in competitions [25]. However, anecdotal evidence shows that some coaches and athletes still doubt the efficiency of SW in building muscle strength and power. The results of this experiment clearly show the superior capacity of SW athletes to produce an explosive upper-body pushing movement compared with a general population of athletes with a similar training volume. Factors that could be responsible for this enhanced efficiency in the SW group include the technique of execution and intramuscular and intermuscular coordination. For example, we can hypothesize that one’s ability to perform a correct scapula fixation—that is, to activate the serratus anterior to stabilize the shoulder blade—results in greater overall stability of the body during the push-ups, less power loss, and therefore a greater jump height. Indeed, [27] showed that push-up variant difficulty influences the level of activation of the serratus anterior. Therefore, over time, SW athletes will likely develop greater activation of this muscle through more advanced push-up exercises. 

Regarding the modality of the execution of push-ups, it was unexpected that, overall, this factor had no significant impact on jump height, and that both groups confounded. Indeed, according to a previous study [1] on the plyometric mechanism, we would have expected to see greater jump heights in PLYO than in CONC and DROP than in CONC and PLYO. Another study [28] went the same way, showing that different indicators of ground reaction forces were different between a countermovement push-up, a simple jump push-up, and a fall push-up. This is because the faster the eccentric phase of the push-up is performed, the greater the ground reaction force should be. 

In our study, the absence of differences in jump height could be due to the fact that, even if the speed and the ground reaction force are greater in PLYO and DROP, this may not necessarily result in greater speed at the moment of takeoff, as suggested by Newton et al. [29]. In this study, the authors analyzed the speed of displacement of a bench press bar during throws. They explained that, even if the stretch–shortening cycle contributed to the beginning phase of the throw, this contribution decreased in the latter portion of the throw. 

Another explanation for the absence of differences in jump height between the modalities of push-ups is that the participants adopted particular landing strategies, as suggested by Dhahbi et al. [21]. The participants could have used a strategy of increased flexion of either the elbows or the shoulders or perhaps the trunk to soften the landing. As a consequence, there might be a slower transition between eccentric and concentric muscle contractions during plyometric and drop push-ups. 

In the present study, the measure of flight time in explosive push-ups was linked to one of the ground reaction force parameters, which was the net vertical force acting on the participant. Indeed, it is possible to estimate the vertical take-off velocity [30] thanks to the method based on the impulse–momentum relationship. Flight time was estimated by placing this velocity into the projectile motion equation. We believe that the measurement of jump height during push-ups, as it was conducted here, presents a practical advantage and is, in reality, similar to what has been undertaken on squat jumps and their variations until this day. Additionally, we think it approaches the validity of experiments that measure ground force reaction parameters with the help of force plates, even if it remains to be proved.

As stated previously, the SW athletes jumped higher on average during the 20 repetitions, but they also maintained a higher jump height during this set than did the CO athletes. This was an unexpected outcome, as it is well known that the higher the maximal force production of an athlete, the faster the decline of this force during a long set [31]. This ability to keep a relatively high jump height in push-ups can be explained by the fact that SW athletes are used to strength–endurance training. Indeed, strength–endurance training consists of keeping a relatively high force production over a given period of time—that is, between 30 and 60 s. This is the type of effort that we could see in some SW competitions (45 s of effort and 45 s of rest) [25,32]. To be more precise, interested readers may refer to the World of Bar Heroes for official regulations in competitions.

In terms of the muscle electrical activity recorded, we observed no significant difference in the filtered EMG signals between our groups and no significant difference between the different modalities of push-ups. Again, the measured data encompassed the normalized maximal amplitude of the RMS of the EMG recorded for each of the three muscles. The absence of differences is not surprising, considering that, when we looked at the individual data, the maximal electrical activity for each participant was reached in CONC, PLYO, or DROP, suggesting that there is not a clear modality of execution of push-up movements that systematically elicits the highest EMG activity. As expected, there was a clear increase in the maximal amplitude of the RMS of the EMG during the sets of 20 repetitions. Indeed, we know that with a fatiguing exercise, the ratio of EMG/force of the effector muscle increases [31,33]. Regarding EMG evolution, we did not observe any significant differences between the groups. Figure 5b–d show that both the SW and CO athletes had approximately the same increase in their muscle electrical activity. Because there was a difference in jump height between groups but no difference in EMG evolution, this would suggest that the ratio of EMG/jump height was different between the groups but not necessarily the ratio of EMG/force, as we know that maximal force output in PU is not necessarily correlated with take-off speed [29]. 

To summarize, the present study contributes three important aspects to the scientific literature: (1) new data on SW, which is a recent sporting discipline that has received very little attention; (2) data on upper-limb plyometric characteristics, which are sidelined in comparison with data on lower limbs; and (3) data on particular bodyweight exercises that are explosive push-ups. Additionally, this is achieved while using the same model as is commonly employed for jumping exercises, such as countermovement jump.

## 5. Conclusions

To conclude, the results obtained with echography and EMG tend to suggest that the greater height reached by SW athletes when performing explosive push-ups can mostly be attributed to peripheral adaptations and to a smaller extent to neuromuscular adaptations. Indeed, two of the four muscles examined had a greater thickness in the SW group, which correlates with a higher cross-sectional area of the muscle [34]. From the EMG measurements, no statistically significant difference was found, although it is worth mentioning that we noted a tendency for the normalized EMG to be higher in the SW group in each modality of push-ups (Figure 4b–d). Finally, one major factor that should be taken into account is the ability to perform explosive push-ups; thus, we could have hypothesized that SW athletes are more efficient in this particular exercise than CO athletes. The lack of difference between the modalities of explosive push-ups, as has been classically observed in the lower limbs, regardless of the level of training of the participants, questions (1) the transfer of plyometric abilities from the lower to the upper limbs and/or (2) the validity of the plyometric ability assessment in the upper limbs by the measurement of the maximal push-up height. Indeed, further investigations are needed to emphasize the exact execution of the movement by tracking the alignment of the body segments, or movement instructions, to determine, for instance, whether countermovement can benefit from jump height in push-up. 

As with many other studies, the present study has some limitations. First, the relatively small sample size may have limited the detection of differences among the groups. Second, further studies should emphasize the validation of push-ups, preferably with kinematic measures, to check whether the shoulders, hips, knees, and ankles are aligned at all times. For interested readers, previous studies have explained how to correctly judge the validity of push-ups [35]. It is important to note that, during the execution of the 20 push-ups, the verbal instructions that were given to encourage the athletes were consistent but not continuous. This could have created small differences in the participants’ cognitive investment between repetitions. Regarding the methodological assessment, echography measurements were performed by only one operator on two images, which may limit the impact of such results. It is usually recommended that measurements be gathered from two different and independent operators to limit the variability of such an operator-dependent measurement. Finally, the validity of the parameter of jump height, estimated by the measure of the flight time when performing the exercise of plyometric push-ups inside the optojump photocell system, remains to be assessed. As explained in the introduction, several studies have proven the validity of the parameters of ground reaction forces measured by force plates during push-up exercises to assess upper-body power [17]. Here, we used optometric cells to measure the jump heights during push-ups and assumed that they were a valid parameter to show the upper-body power of our participants. We believe that this is due to a large body of scientific evidence [36,37,38], and because we devised a very similar experimental protocol for push-up variations to that normally undertaken for lower limbs in squat jump variations.

## 6. Practical Applications

These results suggest that the consistent practice of SW leads to an enhancement of the upper-body muscles’ explosivity. Coaches should be aware that there are exercises and ways of training SW athletes that could be implemented in the training plans of athletes of various other sports if the goal is to increase the overall upper-limb capacity to produce power or, more specifically, to be able to perform a jumping push-up, as is the case in some SW competitions. On the other hand, depending on the sport that the athlete practices, they might not benefit from the plyometric contraction for jumping higher when performing this push-up exercise. Finally, coaches should take into account the quality of execution of push-ups when assessing upper-limb explosive performance, knowing that the technique of execution will have an impact on performance in terms of the maximal jump height that is measured. For example, fixation of the scapula [27] has an important effect on the activation levels of the muscles. This precaution also applies during the training phase, in which coaches should teach the correct technique of execution or at least choose one that is the most appropriate to the athlete and to the goal.

## Data Availability

All raw data can be provided upon request.
